# Evaluation of Electronic–Ionic Transport Properties of a Mg/Zr-Modified LiNi_0.5_Mn_1.5_O_4_ Cathode for Li-Ion Batteries

**DOI:** 10.1021/acsami.3c10480

**Published:** 2023-11-20

**Authors:** Leonardo Balducci, Hamideh Darjazi, Elena Gonzalo, Rosalía Cid, Francisco Bonilla, Francesco Nobili

**Affiliations:** † School of Science and Technology—Chemistry Division, University of Camerino, Via Madonna delle Carceri, ChIP, 62032 Camerino, Italy; ‡ GISEL—Centro di Riferimento Nazionale per i Sistemi di Accumulo Elettrochimico di Energia, INSTM, via G. Giusti 9, 50121 Firenze, Italy; § Group for Applied Materials and Electrochemistry—GAME Lab, Department of Applied Science and Technology—DISAT, Politecnico di Torino, 10129 Torino, Italy; ∥ Centre for Cooperative Research on Alternative Energies (CIC energiGUNE), Basque Research and Technology Alliance (BRTA), Alava Technology Park, Albert Einstein 48, 01510 Vitoria-Gasteiz, Spain

**Keywords:** LNMO, codoping, Mg and Zr, capacity retention, sol−gel synthesis, EIS

## Abstract

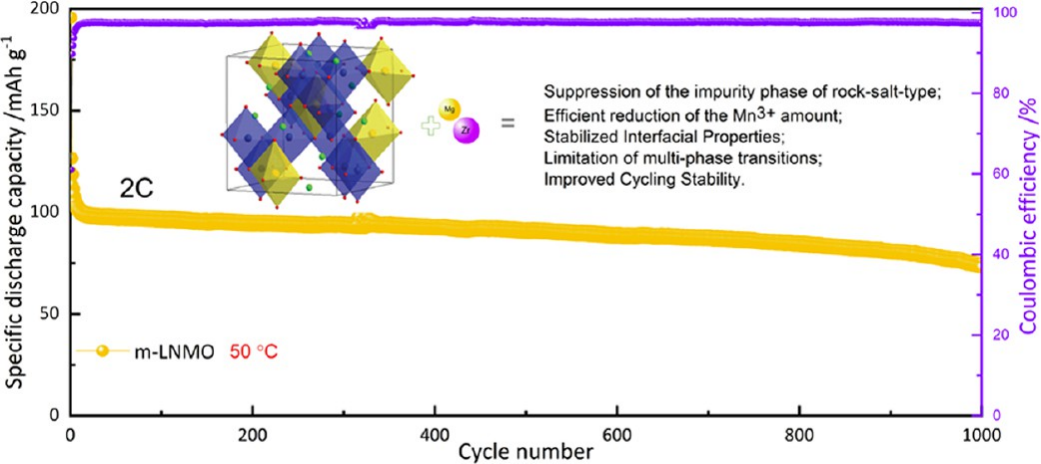

There is an enormous drive for moving toward cathode material research in LIBs due to the proposal of zero-emission electric vehicles together with the restriction of cathode materials in design. LiNi_0.5_Mn_1.5_O_4_ (LNMO) attracts great research interests as high-voltage Co-free cathodes in LIBs. However, a more extensive study is required for LNMO due to its poor electrochemical performance, especially at high temperature, because of the instability of the LNMO interface. Herein, we design structural modifications using Mg and Zr to alleviate the above-mentioned drawbacks by limiting Mn dissolution and tailoring interstitial sites (which are shown by structural and electrochemical characterizations). This strategy enhances the cycle life up to 1000 cycles at both 25 and 50 °C. In addition, a thorough characterization by impedance spectroscopy is applied to give an insight into the electronic and ionic transport properties and the intricate phase transitions occurring upon oxidation and reduction.

## 1 Introduction

In recent years, the pursuit of alternative energy sources has become imperative in order to advance the development of clean energy and highly efficient energy storage technologies. This is driven by the growing demand for sustainable electricity generation and the recognition of the finite supply of fossil fuels.^1,2^ Well-established and efficient energy storage systems are essential to power a range of applications including portable electronic devices, smart grid applications, wearable devices, medical implants, and electrified vehicles.

Li-ion batteries (LIBs) are commonly integrated into these devices due to their high energy density, long cycle life, and relatively good power capability.^3^ However, there are increasing concerns regarding the supply of the cobalt raw material in cathodes for LIBs due to its restricted reserves and price volatility.^4^

Overcoming this concern necessitates the development of “Co-free” cathode materials for LIBs. LiNi_0.5_Mn_1.5_O_4_ (LNMO) is a promising candidate as a high-voltage Co-free cathode for LIBs because of an operating voltage of ∼4.7 V (vs Li^+^/Li) and theoretical specific capacity of around 147 mAh g^–1^.^1^^5^ However, LNMO suffers from capacity decay during prolonged charge/discharge cycling, especially at high temperatures or high C-rates, hindering its large-scale commercialization applications.^6,7^ This drawback is partially associated with the multiple-phase transitions occurring during charge–discharge experiments and the instability of the material structure under high voltage.^8^

Therefore, in order to improve the electrochemical performance of LNMO, an important key is to provide further insights into the reaction pathway and kinetics of the solid-state phase transformation. During the charge, when Li^+^ is extracted from the structure, the LNMO material undergoes “multiple cubic phase transitions”, which strongly affect the lattice parameters and subsequently induce mechanical strains, thus resulting in inferior energy and power density as well as cycling instability.^9^ In addition, under high operating potential, the cationic arrangement is destabilized within the structure in the highly delithiated end-member of the transitions. This behavior is mainly attributed to the departure of Ni from the spinel phase (to a nonoccupied site) to create the rock salt-like phase.^10^ Interestingly, the formation of the rock salt-like phase increases the impedance, inhibiting the electrochemical kinetics and thereby leading to capacity loss upon cycling. On the other hand, at high charge cutoff voltage, cell degradation is principally influenced by a further increase of the parasitic side reactions at high temperature.^11^

In fact, the structural instability of the LNMO spinel can also result from dissolution of Mn^2+^ from the structure into the electrolyte at elevated temperature due to the Jahn–Teller effect upon cycling.^12^

Modification strategies, such as element-based doping, surface coating, and morphology design, have been considered to be efficient approaches to solve these problems and improve the structural stability of LNMO spinel cathode materials. So far, successful reported doping strategies mostly include cation doping in lithium or transition metal sites (e.g., Na^+^,^13^ Sc^3+^,^14^ Al^3+^,^15^ Ti^4+^,^16^ P^5+^,^17^ Cr^3+^,^18^ Zn^2+^^19^), anion doping in oxygen sites (e.g., F^–^,^20^ Cl^–^^21^), or double element-based doping in LNMO (e.g., Y^3+^–Ti^2+^,^22^ Y^3+^–Zn^2+^,^23^ Y^3+^–F^–^,^24^ Li^+^–Br^–^,^25^ Al^3+^–Co^2+^^26^).

Several studies^27−32^ have investigated the impact of the substitution of Mg^2+^ in the LNMO crystal lattice, showing that Mg^2+^ doping enhances the electrical conductivity of the spinel and stabilized the host structure during cycling. Meanwhile, it has been proven that the introduction of Zr^4+^ cations is effective in hindering the formation of nickel oxide-like impurities associated with the existence of Mn^3+^,^33^ stabilizing the structure upon charge–discharge, and ultimately enhancing the cycle life.^34^

In this context, following previous studies on Mg-/Zr- modification of layered cathodes,^35,36^ we pioneer here the processing of Mg/Zr (low-cost elements) codoping of LNMO and present its physicochemical and structural characterization, with the aim of verifying the impact of structural and morphological changes toward charge/discharge behavior and of assessing the improvement of its structural and mechanical stability and, ultimately, the mitigation of its capacity losses.

The sol–gel method is used to prepare cathodes since this technique offers several benefits, including straightforward scalability, cost effectiveness, precise management of the stoichiometric ratios of raw material constituents, and the potential for achieving uniform mixing. Structural and morphological properties are characterized to understand the effects of modification. The charge/discharge performances of cathodes, based on bare and modified LNMO samples, are analyzed to reveal any possible improvement in the stability of the active phase, due to the incorporation of Mg and Zr. Besides, this work also expands the fundamental insights of the electronic and ionic transport kinetics of both LNMO cathodes by electrochemical impedance spectroscopy at different states of charge during lithiation and delithiation, establishing a connection between structural characteristics, bulk conductivity, and cycling performance.

## 2 Experimental Section

### 2.1 Synthesis

Bare and modified cathode powders, labeled as b-LNMO and m-LNMO, respectively, were synthesized by a common synthetic route, corresponding to the citric acid sol–gel method.^36,37^ For m-LNMO, lithium acetate [Li(CH_3_COO)·2H_2_O], nickel acetate [Ni(CH_3_COO)_2_·4H_2_O], and manganese acetate [Mn(CH_3_COO)_2_·4H_2_O], with the addition of magnesium acetate tetrahydrate [Mg(CH_3_COO)_2_·4H_2_O] and zirconium(IV) acetate hydroxide [Zr(OH)_*y*_·(CH_3_COO)_*x*_, *x* + *y* ∼ 4] for the modified powder, were dissolved in distilled water in stoichiometric amounts corresponding to the expected composition “LiNi_0.5_Mn_1.47_Mg_0.025_Zr_0.025_O_4_”. A slight overall (Ni + Mn + dopants) over-stoichiometry was introduced, in order to possibly foster the formation of a Zr oxide coating phase, as previously observed for layered cathodes.^21,22^ Citric acid (CA), as a chelating agent, was then added to the solution, with a molar ratio of CA/total metal cations of 1:1 (all materials from Sigma–Aldrich, purity ≥ 99%). After complete dissolution, the solution was mixed through continuous stirring for 1 day at room temperature. The obtained solution was then slowly heated to 100 °C under continuous stirring until a viscous gel was formed. The gel was dried at 100 °C overnight in a vacuum oven (to further dehydrate), and the residue was ground to a fine powder. A two-step calcination under an air atmosphere was then applied to obtain the final spinel phase. In the first step, the precursor was calcined for 4 h at 480 °C at a heating ramp rate of 2 °C min^–1^ to decompose organic constituents. The second calcination step was carried out for 16 h at 800 °C after a heating ramp rate of 3.7 °C min^–1^ to obtain the final product. The same procedure was adopted for the preparation of b-LNMO, without the addition of Mg and Zr salts.

### 2.2 Chemical, Structural, and Morphological Characterization

The crystal structure and phase ordering of the synthesized compounds were investigated by Raman spectroscopy (Horiba IHR 320, wavelength 532 nm) and X-ray diffraction (XRD) (Bragg–Brentano geometry, Cu Kα, λ = 1.54059 Å). To evaluate the actual lattice parameters, the XRD patterns were analyzed with the FullProf program by profile matching. The electrochemical characteristics of active materials are significantly influenced by morphological attributes, notably, the size distribution of particles. Thus, the particle morphology and elemental composition of the synthesized materials were studied with field emission scanning electron microscopy (SEM) and energy dispersive X-ray elemental analysis (EDX) using an field emission-SEM (FE-SEM) Cambridge Stereoscan 360 electron microscope. In order to gain further insights into the morphology, crystallographic properties, and elemental composition of both samples, an FEI Tecnai G2 transmission electron microscopy (TEM) instrument, equipped with a 200 kV field emission gun and EDX, was utilized. High-resolution TEM (HRTEM) and selected area electron diffraction (SAED) modes on TEM were carried out for local study and comparison of the microstructure of samples. The surface chemical composition of LNMO powders was investigated by means of X-ray photoelectron spectroscopy (XPS) using a nonmonochromatic twin-anode source with Mg Kα (*h*v = 1253.6 eV) operated at 100 W and Al Kα (*h*v = 1486.6 eV) at 150 W. A Phoibos 150 XPS spectrometer with a microchannel plate and delay line detector (SPECS Surface Nano Analysis) installed in a UHV chamber with a base pressure of 5 × 10^–10^ mbar was used for photoelectron collection. The scans were acquired in fixed analyzer transmission mode with 30 eV pass energy and 0.1 eV energy step. The binding energy scale was calibrated by setting the aliphatic C–C bond at 284.8 eV. A Shirley function was employed to simulate the inelastically scattered photoelectron background, and a Voigt profile (30% Gaussian, 70% Lorentzian) was used as the line shape for the photoelectron peaks.

### 2.3 Electrochemical Characterization

The electrodes were prepared using the sample powders (80 wt %), Super P conductive carbon (10 wt %), poly(vinylidene fluoride) (PVDF) (10 wt %), and an appropriate amount of *N*-methyl pyrrolidone (NMP) as a solvent. The slurry was then cast onto an Al foil collector by using a doctor blade set at 150 μm thickness and then dried at 80 °C for 3 h. After drying, circular electrodes were cut, pressed at 2 tons cm^–2^ for 30 s, and vacuum-dried at 120 °C (Buchi Glass Oven) for 12 h. The cathodes were assembled in an Ar-filled glovebox (H_2_O and O_2_ values below 0.1 ppm) for electrochemical measurements using CR2032 coin cells and Swagelok T-type cells. 1 M LiPF_6_ in ethylene carbonate (EC)/dimethyl carbonate (DMC) (1:1 in volume) (Solvionic, 99.9%) was used as an electrolyte, and the glass fiber (Whatman GF/A) was utilized as a separator. Charge/discharge cycles were carried out using a Bio-Logic VMP3 electrochemical workstation in the potential range between 3.5 and 5 V vs Li^+^/Li at different current rates (1C = 147 mA g^–1^, assuming for both electrodes a theoretical capacity of 147 mAh g^–1^). To explore the electrochemical processes taking place at cathodes, cyclic voltammetry (CV) was carried out at a scanning rate of 0.05 mV s^–1^. To provide a comprehensive understanding of the electronic and ionic transport properties upon charge and discharge processes, electrochemical impedance spectroscopy (EIS) measurements have been performed on three-electrode Swagelok T-cells, as a function of the state of charge, in potentiostatic mode, by superimposing an AC perturbation of 10 mV, in the frequency range of 10 mHz–100 kHz, upon the selected bias potentials. Prior to any measurement, 2 h of potentiostatic preconditioning at measurement potential was applied in order to ensure equilibration of the electrodes.

## 3 Results and Discussion

### 3.1 Structural and Morphological Properties

**Figure 1:**
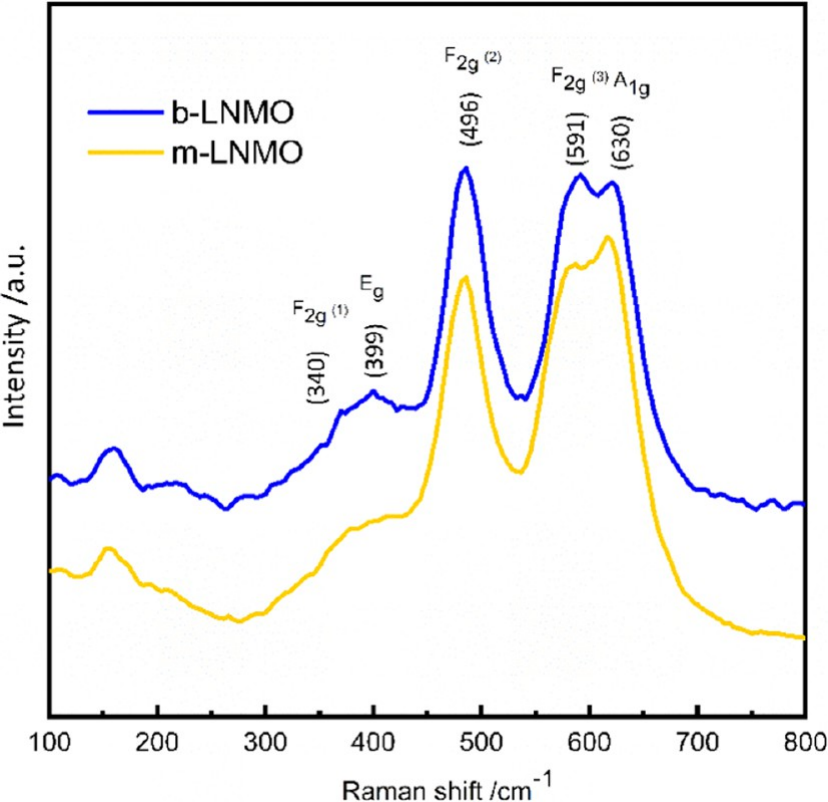
Raman spectral measurement of the b-LNMO and m-LNMO materials.

The LNMO spinel has two types of crystal structures: disordered (space group *Fd*3̅*m*) and ordered (space group *P*4332) phases.^38^ In order to clarify the phase ordering of b-LNMO and m-LNMO samples, Raman spectroscopy was used due to its high sensitivity to crystal symmetry.^17^Figure 1 shows the Raman spectra of the b-LNMO and m-LNMO powders. The spectra show fingerprints of a typical disordered spinel LNMO for both samples, as evidenced by the lack of a few extra peaks around 218 and 237 cm^–1^, which are characteristic of the ordering of the Ni^2+^ and Mn^4+^ in the space group *P*43 32.^39^ The peak at 630 cm^–1^ is attributed to Mn–O stretching mode in the octahedral MnO_6_ groups, belonging to the *A*_1g_ mode, while the peak at 591 cm^–1^ (*F*_2g_^1^) is assigned to the Ni–O band. On the other hand, the peaks at 496 (*F*_2g_^2^) and 399 cm^–1^ (*E*_g_) correspond to the Ni^2+^–O stretching mode in the structure.^40^ The m-LNMO shows a higher intensity of the *A*_1g_ peak, which can be due to shortening of the Li–O1 distance. Since only Mn atoms are connected to O1, the higher intensity of the *A*_1g_ peak is due to an increase in the Mn valence state (higher content of Mn^4+^).^41^ In contrast to the *A*_1g_ mode, the *E*_g_ vibration is assigned to Li–O_2_, where both Mn and Ni are connected to O2. However, mainly, Ni may influence the electron density of O_2_, while Mn is mainly electrochemically inactive.^41^ The m-LNMO shows a lower intensity of *E*_g_ compared to that of b-LNMO, which can be due to the variation of the electronic density of O2, suggesting the increase of Ni–O bond length in m-LNMO.

**Figure 2:**
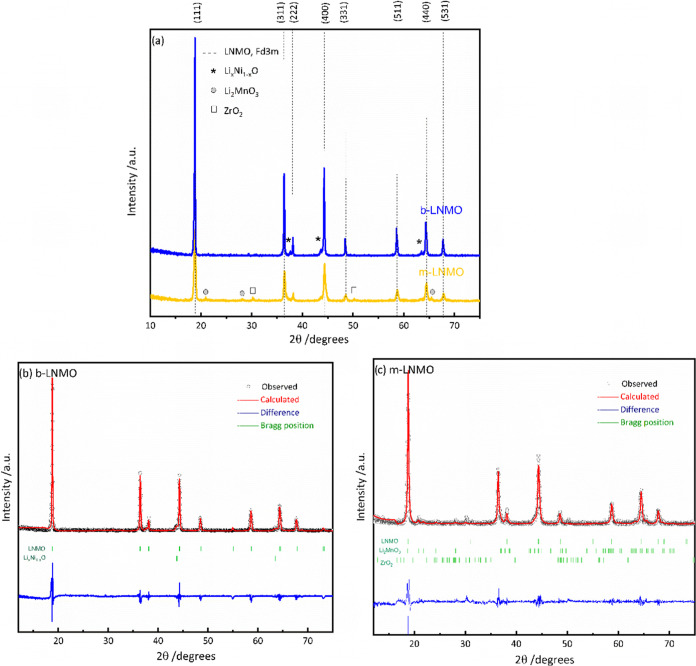
(a) XRD pattern of the b-LNMO and m-LNMO materials. Rietveld refinement results of (b) b-LNMO and (c) m-LNMO.

Figure 2a shows the XRD patterns of the b-LNMO and m-LNMO powders. As can be observed, both patterns correspond to the space group *Fd*3̅*m*,^42^ suggesting that the Mg^2+^ and Zr^4+^ doping has no impact on the inherent cubic spinel structure of the pristine sample. Weak reflections at 2θ around 37, 43, and 64° were detected in the b-LNMO^42^ sample and can be attributed to the Li_*x*_Ni_1–*x*_O impurity phase. This is a common impurity in LNMO materials, which is caused by oxygen loss upon synthesis at high temperatures, and can increase the fading of capacity for spinel LNMO cathode materials.^43^ The formation of an impurity Li_*x*_Ni_1–*x*_O is not evidenced in m-LNMO, suggesting the suppression of the formation of this impurity, possibly by partial substitution of Ni^2+^ by Mg^2+^ or Zr^4+^ ions.^44^ However, traces of the Li_2_MnO_3_ additional phase were detected for the m-LNMO, which is not expected to worsen electrochemical performances.^45^ In order to further investigate the structure of the samples, Rietveld refinement of their XRD pattern was performed using FullProf software, and the results are presented in Table 1 and in Figures 2b,2c. On the basis of the results, the m-LNMO shows a smaller lattice parameter of 8.182(2) Å than that of the b-LNMO (8.170(1) Å). These variations can be correlated with the ionic radius difference among Mn^3+^ (0.645 Å) and Mn^4+^ (0.530 Å),^46,47^ which suggests a slight decrease in the concentration of Mn^3+^ and an increase in the cation ordering (more stable framework) and most probably enhances the cycling performance while reducing the lattice parameter and cell volume.^48,49^

**Table 1:** Rietveld Refinement Results for b-LNMO and m-LNMO Powders

sample	lattice parameter (a) (Å)	cell volume (Å^3^)	*R*_W_ (%)
b-LNMO	8.182(2)	547.78	3.3
m-LNMO	8.170(1)	543.48	3.1

**Figure 3:**
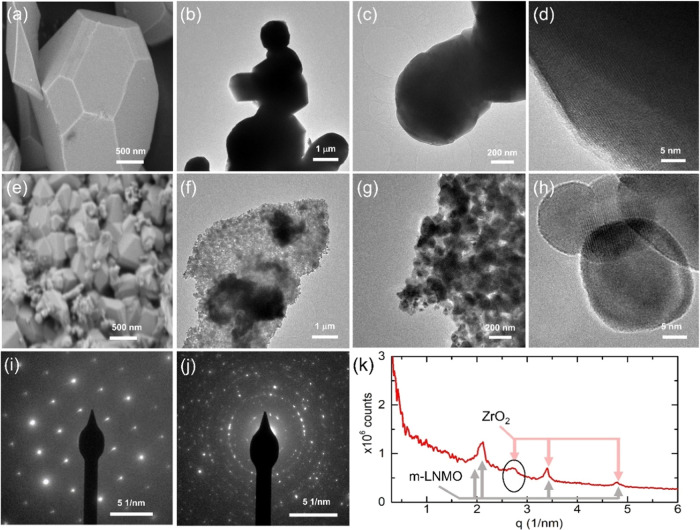
SEM and TEM images of b-LNMO (a–d) and m-LNMO (e–h) powders. Indexed radial integration of the electron diffraction patterns from TEM for the b-LNMO (i) and m-LNMO (j, k) powders.

SEM and high-resolution TEM (HRTEM) images of the powders are shown in Figure 3. The b-LNMO exhibits a morphology of micrometer size (Figure 3a–d), while the m-LNMO is formed by agglomerates of particles in the nanometer range (Figure 3e–h). Further magnification (Figure 3h) clearly shows that the size of m-LNMO particles is on the order of approximately 5–10 nm. A smaller particle size could be beneficial to obtain a higher specific capacity and energy density in spinel cathode materials by shortening the Li-ion diffusion pathway, thus resulting in rapid insertion and extraction of Li ions.^50^ The selected area electron diffraction (SAED) technique was utilized to collect electron diffraction patterns. Panels i and j of Figure 3 correspond to the SAED pattern of b-LNMO and m-LNMO, which is indexed to the planes of (111), (311), (222), (400), (133), (511), (044), and (513). It is worth noting that electron diffraction patterns for a single particle were obtained for b-LNMO, while for the modified sample, electron diffraction patterns for a group of particles were obtained due to the much smaller particle size in comparison to the bare sample. The diffraction reflections of both samples, as expected, match the LNMO spinel structure with the space group *Fd*3̅*m*. Moreover, by looking in deeper detail at the m-LNMO electron diffraction patterns, it is possible to identify a reflection that can be indexed to the <110> ZrO_2_ (Figure 3k), which decorates the active material’s grain. Figure S1 reports the electron diffraction pattern reflection for b-LNMO, which shows no evidence of the previously reported ZrO_2_ peak.

**Figure 4:**
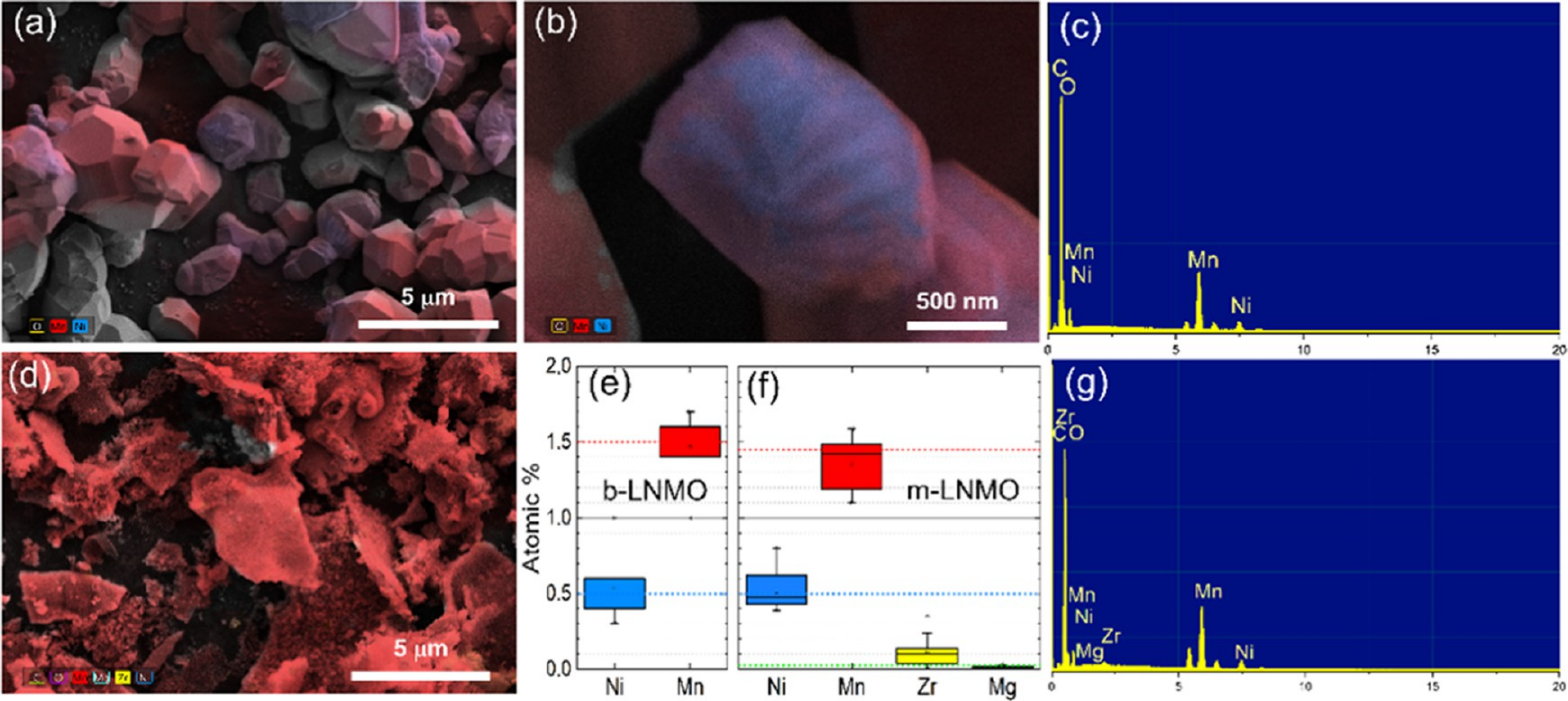
EDX elemental mapping images and corresponding spectrum of b-LNMO (a–c, e) and m-LNMO (d, f, g) powders.

The energy dispersive spectrum and elemental mapping images (Figure 4a–f) indicate that the atomic percentage (atom %) ratios of elements in both bare and modified samples agree with the expected stoichiometries and also show the presence of Zr and Mg in the modified sample. The Ni quantity is around the expected value for both materials, while a larger error bar in Mn determination can be observed in the modified sample compared to the pristine sample (Figure 4e).

**Figure 5:**
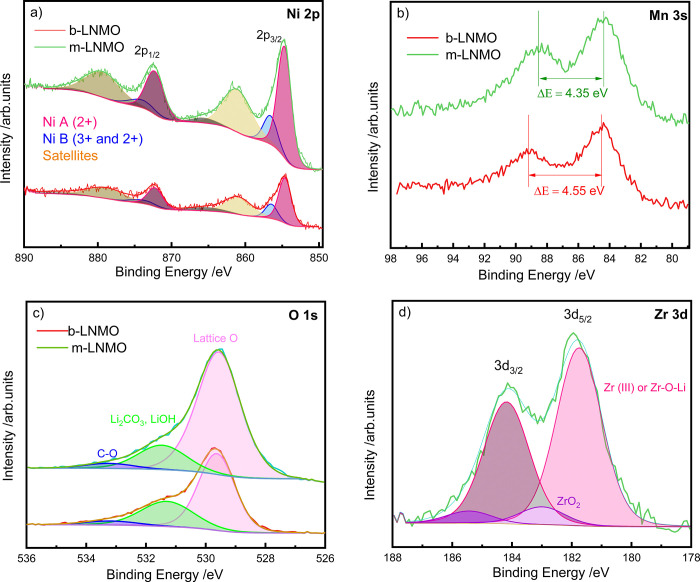
XPS results: Ni 2p, Mn 3s, O 1s, and Zr 3d core levels for b-LNMO and m-LNMO samples.

The chemical states of the LNMO elements were evaluated by XPS. Figure 5a–d shows the typical peaks of Ni 2p, Mn 3s, O 1s, and Zr 3d core levels for both b-LNMO and m-LNMO samples measured with the Mg source. Regions of C 1s and Mn 2p are shown in Figure S2a,b. The C 1s region was extended over 310 eV to include the Mg KLL Auger peak when using the Al source, since all Mg photoelectron peaks overlap with the more intense ones of Mn. Hence, Mg KLL Auger and Zr 3d photoelectron peaks were observed for the m-LNMO samples, indicating the presence of Zr and Mg in the modified sample. The Ni 2p XPS spectra of both samples are quite similar (Figure 5a). Thus, the modification does not have a significant effect on the electronic distribution of Ni, indicating a similar chemical state. We can fit both Ni 2p_3/2_ and Ni 2p_1/2_ spin–orbit split peaks with two components (a main component, Ni A, and a smaller one, Ni B), followed by some satellites. Ni spectra are complex because of multiple splitting effects, and the presence of more than one component in the Ni 2p_3/2_ (Ni 2p_1/2_) peak does not necessarily mean the presence of mixed oxidation states.^51^ In fact, the shape and energy positions of the different features are very similar to those of Ni^2+^ in a standard LNMO sample:^52^ a prominent 2p_3/2_ peak at around 854.6 eV, a 2p_1/2_ one at 872.2 eV, and a main satellite at 861.1 eV. The intensity ratio of Ni B to main Ni A components decreases for m-LNMO (Ni_B_/Ni_A_ = 0.25) versus b-LNMO (Ni_B_/Ni_A_ = 0.30), probably indicating that b-LNMO contains a slight proportion of Ni^3+^, while the modification leads to a purer Ni^2+^ content. The Mn 2p spectra (Figure S2b) are spin–orbit split in 2p_1/2_ and 2p_3/2_ components with binding energies of around 654.3 and 642.7 eV, respectively.^53^ Moreover, the shape and binding energies of the Mn 2p core level are very similar in both samples. However, Mn 2p exhibits significant multiple splitting, and different oxidation states have strong overlapping. Hence, Mn splitting of the 2s signal is usually more informative for evaluating the oxidation state.^54^ As indicated in Figure 5b, the Mn 3s splitting changes from 4.55 eV in b-LNMO to 4.35 eV in m-LNMO. Both values are close to the Mn 3s splitting obtained in a Mn^4+^ reference sample (4.33 eV, compared with 5.30 eV obtained for a Mn^3+^ reference). Nevertheless, the small variation can be correlated to a slight decrease of the Mn^3+^ proportion in the modified sample, which is closer to pure Mn^4+^, in excellent agreement with the results obtained by XRD. The XPS spectra (Figure 5c) of both samples show a main peak at 529.6 eV, related to the LNMO lattice oxygen, and extra peaks at around 531.5 and 533.2 eV, corresponding to surface species, mainly carbonates and/or hydroxides. Figure 5d shows the spin–orbit split Zr 3d core level for m-LMNO in the doublet formed by 3d_5/3_ and 3d_3/2_ components. A proper fitting requires the use of two doublets, meaning that two chemical environments are found for Zr. The smaller one with a 3d_5/3_ component at 183.0 eV can be related to some residual ZrO_2_, while the main one with a 3d_5/3_ peak at lower binding energy (181.8 eV) could be ascribed to the presence of Zr^3+^ within the doped LNMO (or Zr^4+^ in a less electronegative environment because of the presence of Li^+^). This Zr doping can be thought to substitute mainly for Mn^3+^ so that Mn in the m-LNMO is closer to pure 4+ as previously discussed. In agreement with the preferential substitution of Mn, the Mn/Ni ratio substantially decreases from 3.4 in b-LMNO to 2.0 in the m-LNMO. The calculated ratio of Zr/Ni is 0.25 in the modified sample. Although the presence of Mg is confirmed in this sample by the KLL Auger line (Figure S2a), its concentration cannot be properly quantified due to the overlap of Mg photoelectron peaks with those of Mn and only an upper limit can be obtained (Mg/Ni ≤ 0.1).

### 3.2 Electrochemical Characterization

**Figure 6:**
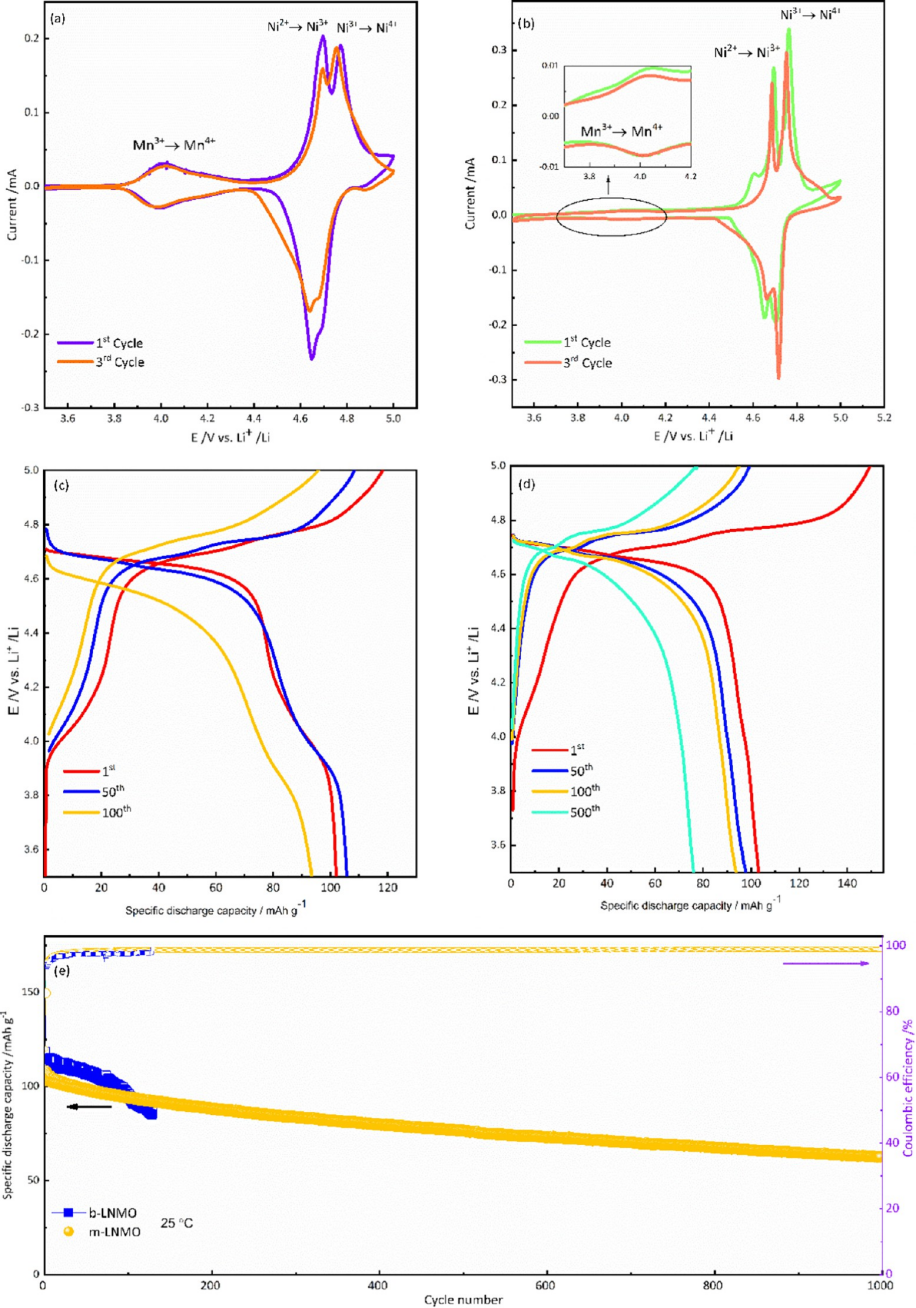
Electrochemical performance comparison between 3.5 and 5 V at 25 °C. Cyclic voltammetry of b-LNMO (a) and m-LNMO (b) samples, at a scan rate of 0.5 mV s^–1^; E vs Q galvanostatic profiles of b-LNMO (c) and m-LNMO (d) electrodes. Cycling performance of b-LNMO and m-LNMO (e). Cycling rate at 1C.

To explore the electrochemical processes occurring on both cathodes during charge/discharge, cyclic voltammetry was performed at a scan rate of 0.05 mV s^–1^ in the range of 3.5–5 V vs Li^+^/Li at 25 °C, as shown in Figure 6a,b. Both cathodes show two oxidation peaks located at ∼4.69 and 4.75 V and corresponding reduction peaks at ∼4.64 and 4.69 V. These redox peaks are the redox reactions between Ni^2+^/Ni^3+^ and Ni^3+^/Ni^4+^ and are the main electrochemical reactions in LNMO materials.^44^ It should be noted that the voltammetric peaks of m-LNMO are sharper than the corresponding ones of b-LNMO, suggesting a lower bulk resistance of the modified material as a consequence of doping. The reversible plateau of about 4.0 V is related to the Mn^3+^/Mn^4+^ redox couple,^38−40^ highlighting the presence of Mn^3+^ and the disorder structure of LNMO in both samples. However, this feature is much less relevant in m-LNMO than in b-LNMO, confirming the higher average oxidation state of Mn (and thus a lower amount of Mn^3+^) for the modified sample. Besides, during the cathodic scan, a peak at ∼4.6 V vs Li^+^/Li can be observed in m-LNMO, which corresponds to the oxidation of the Li_2_MnO_3_ phase as evidenced by XRD analysis. After the first oxidation, this peak is no longer observed, suggesting the irreversible electrochemical activity of Li_2_MnO_3_.^45^

**Figure 7:**
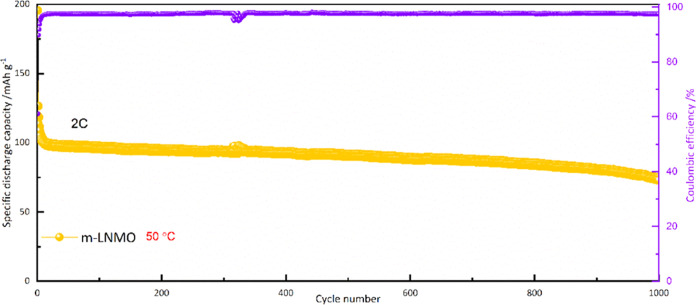
Cycling performance of m-LNMO between 3.5 and 5 V at 50 °C. Cycling rate of 2C.

**Figure 8:**
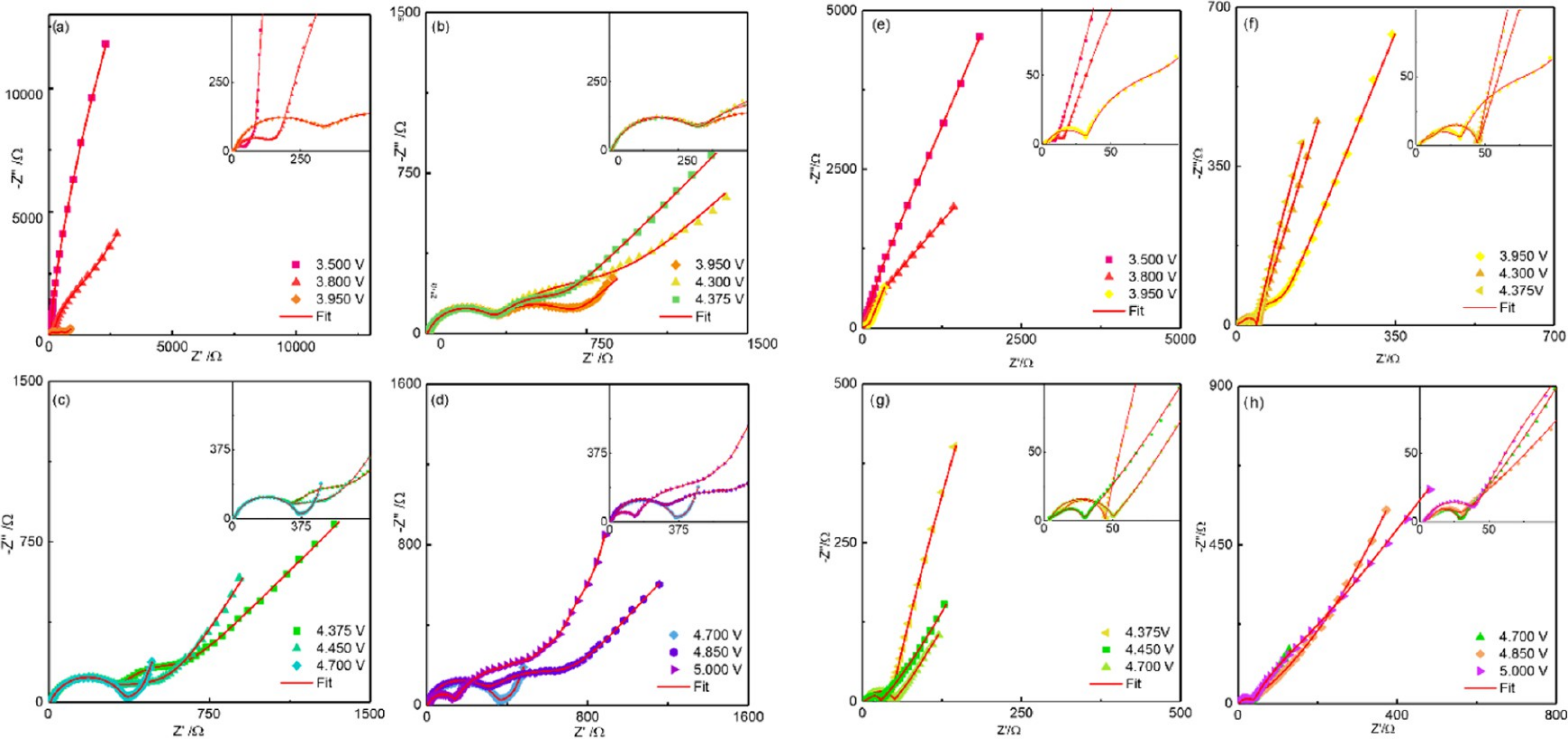
Selected Nyquist plots for b-LNMO (a–d) and m-LNMO (e–h) upon the initial Li^+^ extraction.

In order to evaluate the impact of the modification on the cycling performance, two-electrode cells were tested at 1C charge/discharge rate, in a voltage range from 3.5 to 5.0 V vs Li^+^/Li at 25 °C. The galvanostatic charge/discharge E vs Q profiles of b-LNMO (Figure 6c) and m-LNMO (Figure 6d) show a potential plateau at around 4.7 V along with a potential plateau at about 4.0 V, consistent with the CV peaks of Ni^2+^/Ni^4+^ and Mn^3+^/Mn^4+^ redox processes, respectively. As observed, the LNMO modification remarkably suppresses the redox process at ∼4.0 V because of the lower Mn^3+^ content. In Figure 6e, the b-LNMO and m-LNMO show similar first-cycle discharge capacity values of 102.4 and 102.8 mAh g^–1^ at 1C, with initial Coulombic efficiency (CE) values of 86.2 and 68.7%, respectively. The lower initial CE of m-LNMO can be tentatively attributed to two kinds of irreversible processes: (i) the irreversible oxidation of Mn^3+^ in the Li_2_MnO_3_ phase and (ii) irreversible formation of the cathode electrolyte interphase (CEI), occurring at a larger extent for m-LNMO because of the larger interfacial area associated with the smaller particle size. With regard to the long-term cycle life, b-LNMO and m-LNMO show similar behaviors upon the initial 100 cycles. Subsequently, the b-LNMO cell undergoes a fast capacity decay, ultimately resulting in cell failure after around 130 cycles. On the contrary, the m-LNMO electrode shows much better performances, with an excellent capacity retention of 80% after 400 cycles and 60.7% after 1000 cycles. In addition, for the m-LNMO electrode, CE is held higher than 99.4% up to the 1000th cycle, while for b-LNMO, the CE values never exceed 97.8% until cell failure. In order to verify electrode stability, the cycling behavior of cathodes has been tested in more demanding conditions, such as at *T* = 50 °C and 2C charge/discharge (3.5 V ≤ *E* ≤ 5 V). As shown in Figure 7, the initial discharge capacity of m-LNMO is 119.4 mAh g^–1^, while minor irreversibility can be observed up to the eighth cycle. Then, the reversible capacity stabilizes at 100.5 mAh g^–1^, with a coulombic efficiency of 97.5% and cycling stability of ∼72.8% after 1000 cycles (comparable or even superior to those reported in the literature, see Table S1). It is important to note that b-LNMO exhibits a significant drop in specific capacity when cycled at 50 °C (Figure S3). The superior capacity retention of m-LNMO, especially at higher temperature and C-rate, can be explained by the synergistic actions of Mg and Zr modification toward (i) suppression of the impurity phase of rock salt-type Li_*x*_Ni_1–*x*_O, by replacement of Ni^2+^ by Mg^2+^ or Zr^4+^ ions;^36^ (ii) efficient reduction of the Mn^3+^ amount (by partial substitution with dopants (possibly Zr^3+^, as revealed by XPS)) with subsequent mitigation of the Jahn–Teller distortions and decrease of the interfacial side reactions, which ultimately results in maintaining the stability of the structure.^25^ In addition, the decreased Mn^3+^ content results in less Mn dissolution, which is otherwise promoted by disproportion 2Mn^3+^ → Mn^4+^ + Mn^2+^, especially at high temperatures;^55^ (iii) reduction of the grain size, which results in shorter and faster Li diffusion in the solid;^50^ and (iv) generation of further diffusion pathways within the electrode structure bulk by ZrO_2_ clusters.^56^ In order to investigate the impact of reversible and irreversible structural transitions on the kinetics of the charge/discharge processes, EIS measurements at several states of charge and potentials have been performed during the first charge/discharge cycle for both b-LNMO and m-LNMO electrodes. Staircase potentiostatic electrochemical impedance spectroscopy (SPEIS) has been applied in the potential range of 3.5–5 V with 25 mV steps, both during oxidation and reduction. Figure 8a–d shows Nyquist plots of the b-LNMO electrode at selected potentials during the first charge. Figure 8e,f shows the corresponding Nyquist plots for m-LNMO.

All of the spectra show some common features, which may appear more or less pronounced depending on the state of charge and the sample investigated. Based on previous EIS investigations of LIB cathode materials,^36,57^ the following contributions to overall impedance can be recognized in the Nyquist plots and assigned to specific steps in the electronic/ionic transport and redox processes, from high to low frequencies: (i) an intercept with the real axis, which describes a pure resistive behavior and can be attributed to electrolyte resistance; (ii), (iii) two convoluted arcs in the high-to-middle frequencies, where the higher frequency one is much smaller, nevertheless, can be observed clearly, for instance, in the inset in panel 8g, and can be related, respectively, to migration/accumulation of Li^+^ at the CEI and charge transfer resistance/charge accumulation at the electrical double layer; (iv) a low-frequency arc, which is the most relevant feature at low potentials and can be assigned to bulk electronic resistance of the material^49^ and intragrain charge accumulation at crystallite boundaries;^58^ and (v) a 45 deg dispersion, bending toward a vertical line, which commonly describes diffusion toward a blocking electrode. The most relevant trend that can be observed through the impedance dispersions is variation of the diameter of the low-frequency semicircle, which undergoes contraction and expansion for both electrodes, even if with different paths, upon charge.

For the b-LNMO cathode, the large low-frequency arc (iv) related to electronic resistance contracts in the potential range of 3.500–3.950 V (Figure 8a), partially expands from 3.950 to 4.375 V (Figure 8b), contracts again from 4.375 to 4.700 V (Figure 8c), and finally expands, even if not to the initial width, from 4.700 to 5.000 V (Figure 8d). This behavior highlights multiple, progressive, partly reversible transitions between insulating and conducting phases by structural rearrangements.^49^ On the other side, the m-LNMO cathode exhibits a monotonous contraction of the low-frequency arc from 3.500 to 4.700 V (Figure 8e–g), with a final, partial expansion from 4.700 to 5.00 V (Figure 8h). This behavior underlines that the Mg/Zr-modified cathode is less prone to structural rearrangements. The two different behaviors are reversed during the subsequent discharge of b-LNMO and m-LNMO cathodes, as shown in Figure S4, where the same trends can be observed in almost overlapped potential regions but in the opposite direction. This evidences that the rearrangement pathways resulting in insulator-to-conducting transition properties are mostly reversible.

With regard to the semicircles at mid and high frequency, their diameters face only limited oscillations, demonstrating that CEI- and charge transfer-related processes are much less dependent on the state of charge.

The equivalent circuit method has been applied to quantitatively describe the evolution of the ionic and electronic transport properties in the investigated potential range. The following equivalent circuit, written in Boukamp’s notation,^59^ has been used to simulate the experimental data, *R*_sol_(*R*_CEI_*C*_CEI_)(*R*_ct_*C*_dl_)([*R*_elec_*W*]*C*_elec_) *C*_i_, where *R*_sol_ is the electrolyte resistance, *R*_CEI_ and *C*_CEI_ are resistance and capacitance of the passivation layer, respectively, *R*_ct_ and *C*_dl_ are charge transfer resistance and double-layer capacitance, respectively, *R*_elec_ and *C*_elec_ are bulk electronic resistance of the active material and capacitance due to intercrystallite charge accumulation, respectively, *W* is Warburg impedance related to diffusion, and *C*_i_ is differential intercalation capacity (Figure S5). To take into account any deviation from ideal behavior arising from electrodes’ inhomogeneity or roughness, all the *C* elements have been replaced by constant-phase elements *Q* in the fitting procedure, which has been carried out by using RelaxIS software by rhd Instruments.

**Figure 9:**
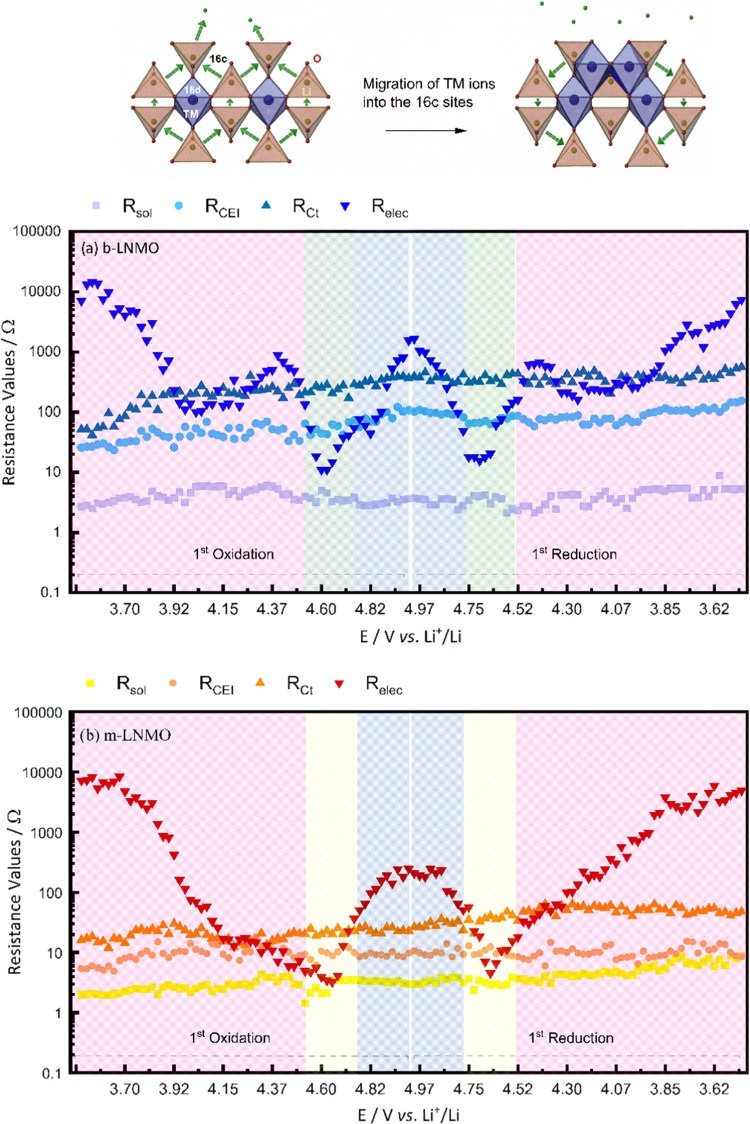
Calculated values of *R*_sol_, *R*_CEI_, *R*_Ct_, and *R*_elec_ for (a) b-LNMO and (b) m-LNMO cathodes.

Figure 9 shows the calculated resistance values for *R*_sol_, *R*_CEI_, *R*_ct_, and *R*_elec_ in the 3.500–5.000 V range (60 values each upon charge and 60 upon discharge for both cells). Consistent with the Nyquist plots, for b-LNMO, the values of *R*_elec_ continuously decrease during charge from 3.500 to 3.950 V; then, the values increase from 3.950 to 4.375 V of about 1 order of magnitude, to decrease again from 4.375 to 4.700 V. This behavior can be explained with the overlap of two concurrent and counteracting phenomena: an increase of material conductivity as Li^+^ is progressively extracted from the structure,^60^ which is partly counteracted in a limited potential region (3.950 ≤ *E* ≤ 4.375 V) by structural rearrangements associated with Mn^3+^ to Mn^4+^ oxidation, which leads to the coexistence of multiple cubic phases, resulting in lattice mismatch, structural stress, and, ultimately, entangled Li^+^ and e^–^ diffusion in the structure.^57^ As the result of the two concurrent phenomena, the *R*_elec_ reaches the minimum value at 4.700 V; then, the values significantly increase by about 2 orders of magnitude as a consequence of the rock salt-like phase formation, where some transition metal (TM) ions migrate from 16d to 16c sites.^9,10^ The occupation of the 16c sites by TM ions at the end of the charging process may block Li^+^ diffusion pathways through the active grains, resulting in limitations to the concurrent electron mobility.

With regard to the m-LNMO cathode, the *R*_elec_ values continuously decrease upon charge from 3.500 to 4.700 V. This suggests that the electronic conductivity variation in this potential range is only controlled by the Li^+^ extraction, resulting in a monotonous decrease of *R*_elec_, while the reduced Mn^3+^ content due to the doping suppresses the rearrangement to multiple cubic phases and the subsequent lattice mismatch, which was at the origin of the partial conductivity decrease for b-LNMO. Above 4.700 V, also, the m-LNMO electrode undergoes the rock salt-like phase formation, resulting in the final *R*_elec_ increase up to 5.000 V. Nevertheless, the increase is slightly limited (∼300 Ω for m-LNMO vs ∼1000 Ω for b-LNMO), demonstrating stabilization of the modified cathode material toward this phase transformation as well. During the discharge, opposite trends of *R*_elec_ values are evidenced for both cathodes, confirming the reversibility of their insulating/conducting properties.

With regard to *R*_ct_, *R*_CEI_, and *R*_sol_, for both electrodes, the values show slightly increasing trends, suggesting initial evolution of the interfaces. However, for the m-LNMO, *R*_ct_ and *R*_CEI_ values are kept about 1 order of magnitude lower than for the b-LNMO, demonstrating enhanced interfacial stability for the modified cathode.

## 4 Conclusions

In conclusion, we have explored the effect of dual doping on the stability and performance of the high-voltage LNMO cathode material. We present a new approach to increase the lifetime of the LNMO material by combined Mg and ZrO_2_ nanocluster doping using one-pot sol–gel synthesis. To the best of our knowledge, this is the first time that such a facile approach has been successfully applied to an LNMO cathode. The presence of the Mg atomic doping and the ZrO_2_ nanocluster has been confirmed by the various structural techniques exploited.

The electrochemical test showed an increased cycle life and capacity retention of up to 1000 cycles at both 25 and 50 °C. It has been demonstrated that several modifications induced by Mg/Zr dual doping concur in enhancing the electrochemical behavior.

First, the evaluation of interfacial process resistance, done by electrochemical impedance spectroscopy, showed a decreased *R*_CEI_ and *R*_Ct_ compared to the bare LNMO. This suggests that the formation of a thicker and more stable CEI, also involving surface-decorating ZrO_2_, prevents undesirable side reactions within the electrode surface. This allowed us to successfully mitigate the Mn dissolution, resulting in stable performance and highly improved capacity retention.

Besides, the mitigation of bulk *R*_elec_ changes for the m-LNMO electrode suggests that the structural modifications, due to the dual Mg/Zr doping, effectively suppress or limit the detrimental phase transitions, contributing to the excellent capacity retention and high-rate performances for the LNMO cathode.

Finally, in addition to demonstrating the improvement of the electrochemical behavior of the modified LNMO cathode, the presented results open the possibility of applying these facile and successful modifications to other cathode materials in the quest for Co-free electrodes.

For these reasons, we think that the presented results may give a significant contribution to advancements of the state-of-the-art cathode materials for LIBs, paving the way to more stable, performant, sustainable, and durable batteries.

## Supporting Information Available

Indexed radial integration from TEM for b-LNMO; XPS analysis for C 1s and Mn 2p for both b-LNMO and m-LNMO; cycling performance of b-LNMO at 50 °C; Nyquist plots for both b-LNMO and m-LNMO during Li^+^ insertion; equivalent circuit used to simulate the data; and selected literature overview of the performance of the LNMO cathode (PDF)
