# Supplementary material for: Evaluation of Electronic–Ionic Transport Properties of a Mg/Zr-Modified LiNi0.5Mn1.5O4 Cathode for Li-Ion Batteries
Source: ACS Appl Mater Interfaces. 2023 Nov 20;15(48):55620–32. doi: 10.1021/acsami.3c10480 (PMC10711703; doi:10.1021/acsami.3c10480)
Supplement: Supplementary file 1 — am3c10480_si_001.pdf [file am3c10480_si_001.pdf]

Evaluation of Electronic-Ionic Transport Properties of a Mg-/Zr-Modified $LiNi_{0.5}Mn_{1.5}O_4$ Cathode for Li-Ion Batteries.

Leonardo Balducci[a*], Hamideh Darjazi[a,b,c,**], Elena Gonzalo[d], Rosalía Cid[d], Francisco Bonilla[d], Francesco Nobili[a,b]

[a]*School of Science and Technology - Chemistry Division, University of Camerino, Via Madonna delle Carceri, ChIP, 62032 Camerino, Italy.*
[b]*GISEL—Centro di Riferimento Nazionale per i Sistemi di Accumulo Elettrochimico di Energia, INSTM, via G. Giusti 9, 50121 Firenze, Italy.*
[c]*Present address: Group for Applied Materials and Electrochemistry - GAME Lab, Department of Applied Science and Technology - DISAT, Politecnico di Torino, Torino, 10129, Italy.*
[d]*Centre for Cooperative Research on Alternative Energies (CIC energiGUNE), Basque Research and Technology Alliance (BRTA), Alava Technology Park, Albert Einstein 48, 01510 Vitoria-Gasteiz, Spain.*
*Corresponding authors: * leonardo.balducci@unicam.it and **hamideh.darjazi@polito.it.*

Supporting Information

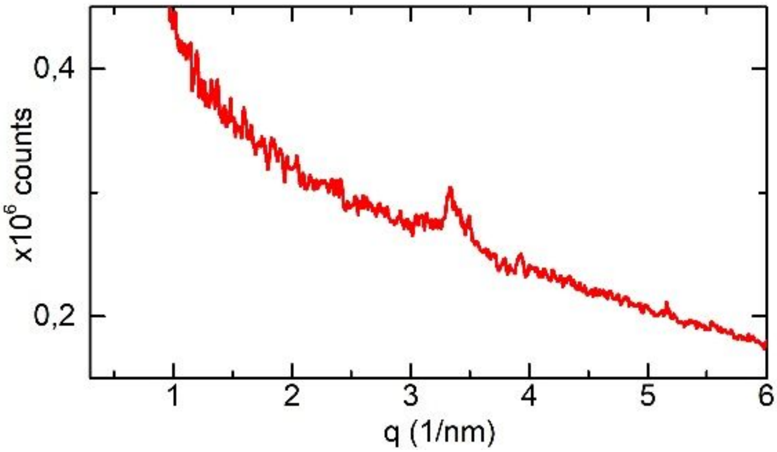

Figure S1: Indexed radial integration of the electron diffraction patterns from TEM for the b-LNMO powders.

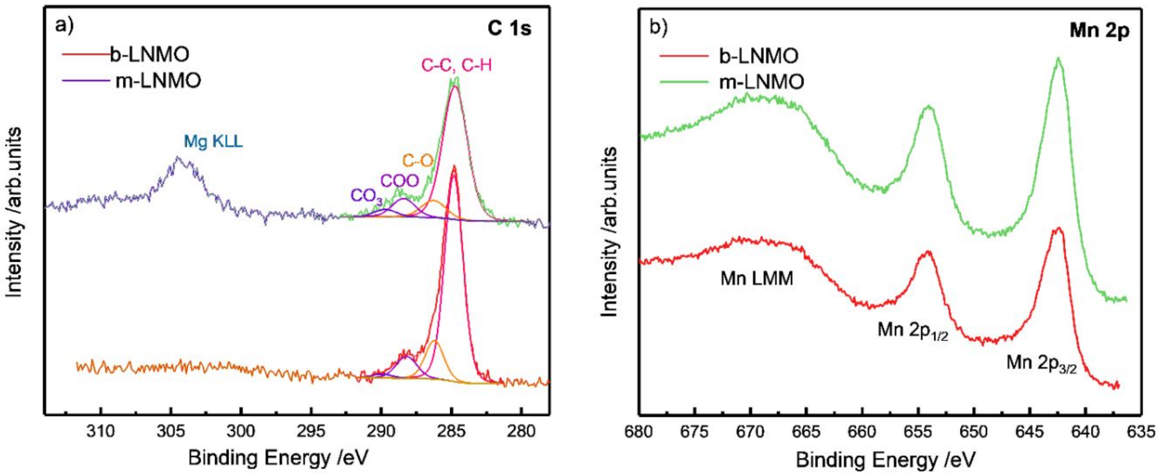

Figure S2: XPS results: (a) C 1s and (b) Mn 2p core levels for b-LNMO and m-LNMO samples, respectively.

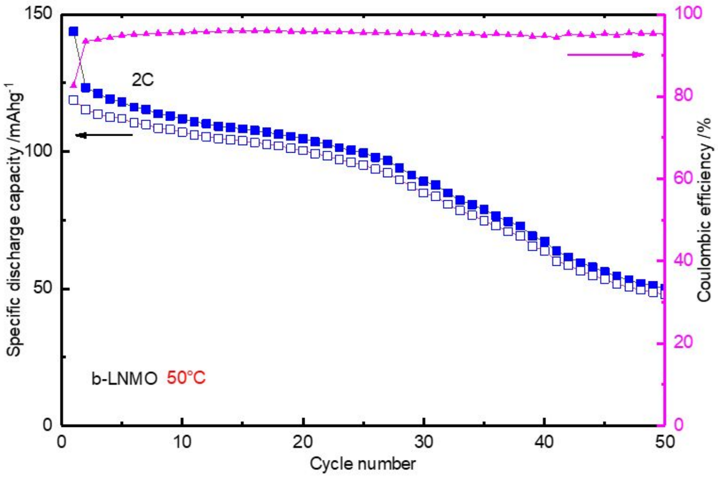

Figure S3. Cycling performance of b-LNMO between 3.5 V to 5 V at 50 °C. Cycling rate 2C.

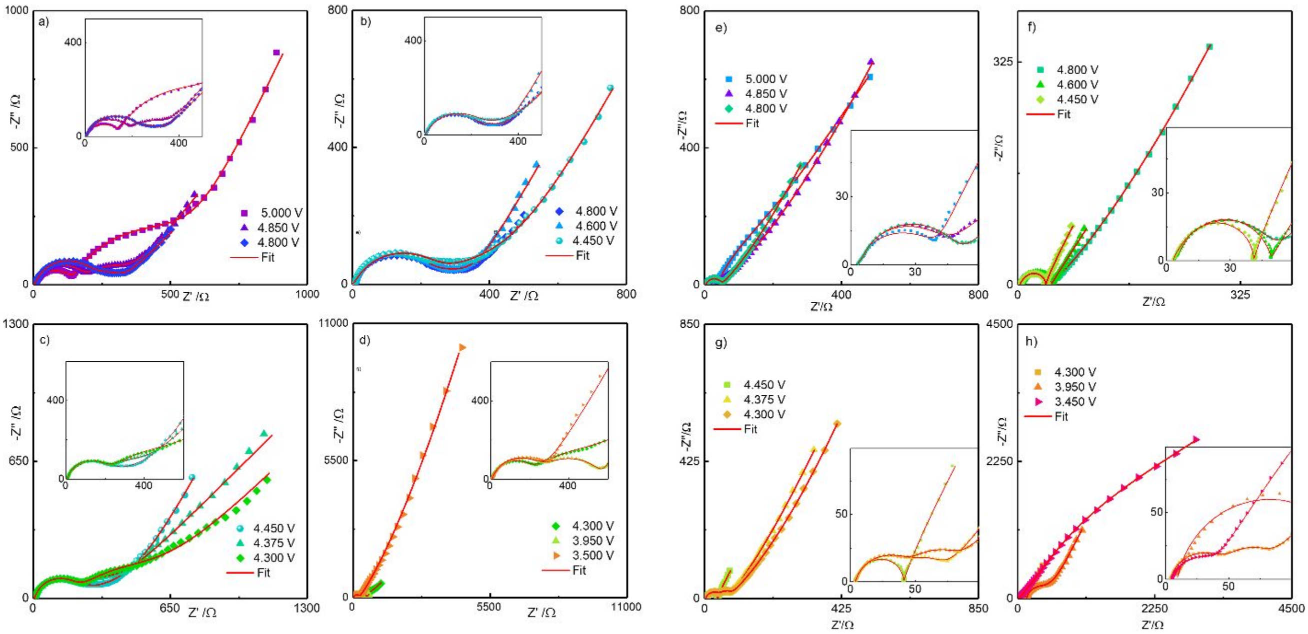

Figure S4. The selected Nyquist plots for b-LNMO (a-d) and m-LNMO (e-h) upon the initial Li^+ insertion.

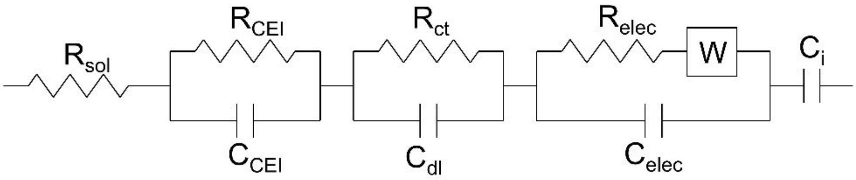

Figure S5. Equivalent circuit used to simulate the data.

Table S1. Selected literature overview of the performance of LNMO cathode.

Precursor	***Capacity retention***	***Cycle number***	***Current density***	***Refs.***
Sr^{2+} doped LNMO	86.63%	500	1C, 25°C	1
Interstitial site controlled LNMO	82%	900	1C, 25°C	2
Ce-GO coated LNMO	95.3%	100	C/10, 25°C	3
HCB coated LNMO	95%	100	1C, 25°C	4
LTO doped LNMO	98.8%	100	2C, 55°C	5
Y^{3+}-F^{-} co-doped LNMO	96.8%	200	1C, 25°C	6
V^{+5} doped LNMO	87.9%	200	4C, 25°C	7
Zn-enriched LNMO	87%	100	C/3, 25°C	8
LATP coated LNMO	84.92%	300	1C, 25°C	9
Fluorine-doped LNMO	81.5%	100	C/5, 55°C	10
Surface modified LNMO	95.1%	150	1C, 25°C	11
Zn-Y co-doped LNMO	79.6%	120	1C, 55°C	12
Li^{+}-Br^{-} co-doped LNMO	95.3%	270	1C, 25°C	13
LNMO coated with CeO_2 and wrapped in graphene	95.3%	100	C/10, 25°C	14
SmF_3-coated LNMO	81.3%	200	1C, 25°C	15
Mg/Zr modified LNMO	72.8%<br>80% &<br>60.7%	1000<br>400 and 1000	2C, 50°C<br>1C, 25°C	**This work**

References

1. Ji, X. *et al.* In situ Sr^{2+}-doped spinel $LiNi_{0.5}Mn_{1.5}O_4$ cathode material for Li-ion batteries with high electrochemical performance and its impact on morphology. *Ceram Int* **47**, 32043–32052 (2021).

2. Han, Y. *et al.* Suppressed phase separation in spinel $LiNi_{0.5}Mn_{1.5}O_4$ cathode via interstitial sites modulation. *Nano Energy* **91**, (2022).

3. Qureshi, Z. A. *et al.* Influence of graphene wrapped-cerium oxide coating on spherical $LiNi_{0.5}Mn_{1.5}O_4$ particles as cathode in high-voltage lithium-ion batteries. *J Alloys Compd* **920**, (2022).

4. Liu, Y. H., Chen, W. C., Hsueh, C. H. & Hsu, C. L. Elucidating the function of modified carbon blacks in high-voltage lithium-ion batteries: impact on electrolyte decomposition. *Mater Today Chem* **25**, (2022).

5. Gao, C., Liu, H., Bi, S., Fan, S. & Xie, Y. Unveiling the role of Ti substitution in improving safety of high voltage $LiNi_{0.5}Mn_{1.5-x}Ti_xO_4$ cathode material by ameliorating Structure-stability and enhancing Elevated-temperature properties. *Appl Surf Sci* **599**, (2022).

6. Lin, F. *et al.* The action of Y-F co-doping in $LiNi_{0.5}Mn_{1.5}O_4$ positive electrode materials. *Powder Technol* **409**, (2022).

7. Hsu, S. C. *et al.* Synergistic effect of doping and surface engineering on $LiNi_{0.5}Mn_{1.5}O_4$ and its application as a high-performance cathode material for Li-ion batteries. *Ceram Int* **48**, 27859–27869 (2022).

8. Garg, S. *et al.* Zn-enriched cathode layer interface via atomic surface reduction of $LiNi_{0.5}Mn_{1.5}O_4$: Computational and experimental insights. *J Power Sources* **569**, (2023).

9. Kang, H. *et al.* Phosphazene based LATP precursor for a CEI coating layer on high voltage $LiNi_{0.5}Mn_{1.5}O_4$ cathode with improved cycling durability. *Mater Chem Phys* **290**, 126492 (2022).

10. Shih, C. P. *et al.* Spray-drying synthesis of fluorine-doped $LiNi_{0.5}Mn_{1.5}O_4$ as high-voltage cathodes for lithium-ion batteries. *J Alloys Compd* **932**, 167641 (2023).

11. Huang, W. *et al.* Electrochimica Acta Bifunctional urea surface-modified high voltage $LiNi_{0.5}Mn_{1.5}O_4$ cathode for enhanced electrochemical performance. *Electrochim Acta* **458**, 142525 (2023).

12. Chen, T. *et al.* Zn-Y co-doped $LiNi_{0.5}Mn_{1.5}O_4$ cathode materials with high electrochemical performance. *J Alloys Compd* **941**, 168825 (2023).

13. Mu, J. *et al.* Exploring the synergistic effect of Li+ and Br− co-doping on improving the microstructural and electrochemical performances of $LiNi_{0.5}Mn_{1.5}O_4$ cathode materials. *J Taiwan Inst Chem Eng* **138**, 104437 (2022).

14. Qureshi, Z. A. *et al.* Influence of graphene wrapped-cerium oxide coating on spherical $LiNi_{0.5}Mn_{1.5}O_4$ particles as cathode in high-voltage lithium-ion batteries. *J Alloys Compd* **920**, 165989 (2022).

15. Lian, Z. *et al.* Improving electrochemical performance of $LiNi_{0.5}Mn_{1.5}O_4$ positive electrodes via regulated cathode electrolyte interphase. *Chemical Engineering Journal Advances* **16**, 100524 (2023).
